# Comparing homologous microscopic sections from multiple embryos using HREM

**DOI:** 10.1016/j.ydbio.2016.05.011

**Published:** 2016-07-01

**Authors:** R. Mark Henkelman, Miriam Friedel, Jason P. Lerch, Robert Wilson, Timothy Mohun

**Affiliations:** a Mouse Imaging Centre (MICe), Hospital for Sick Children, University of Toronto, Toronto, Canada; b The Francis Crick Institute Mill Hill Laboratory, London, UK

## Abstract

• 3D HREM embryo images can be registered.
• Homologous microscopic sections can be obtained from multiple embryos.
• Anatomical phenotypes can be analyzed by computer.

## 1 Introduction

Developmental biologists frequently want to compare morphological and functional characteristics over groups of individuals. Mechanisms of development are frequently studied by modifying the genetic or regulatory controls in one group and comparing it with a wild type group. Such comparisons can be made at multiple scales. In mouse embryos, it is quite common to compare whole mounts often enhanced with LacZ expression. Gene expression patterns using fluorescence microscopy with several reporter fluorescent proteins knocked in are also very common. However, quite frequently, one wants to make comparisons at a cellular level with high-resolution microscopy. Attempting to obtain equivalent sections from fixed and cut blocks is well known to be problematic. No matter what the care in mounting specimens and in prescribing angulations of the cuts with the microtome, one very seldom ends up with histological sections which are directly comparable over several different individuals. Sectioning of multiple adjacent sections helps fill in the need for a three-dimensional representation; however, it remains an exercise in three-dimensional conceptual gymnastics to compare multiple sections from several individuals and find equivalent regions of anatomy. Therefore, in comparing histology, one is very frequently left with an ambiguity as to whether differences in anatomical pattern or in fluorescence expression are really significant differences between individuals or whether they simply arise from the differences of location in the histological section across the different samples. Examining multiple individuals for both the control and manipulated group helps build confidence that differences, which are recognized multiple times, are likely to be real and systematic differences. However, if there is partial penetrance, ambiguity remains and can lead to even greater uncertainty.

Thus it would be ideal, if it were possible, to obtain exactly equivalent microscopic slices of anatomy from groups of individuals that could be inspected side by side to identify with confidence any differences in anatomy or gene expression.

## 2 Results and discussion

**Fig. 1:**
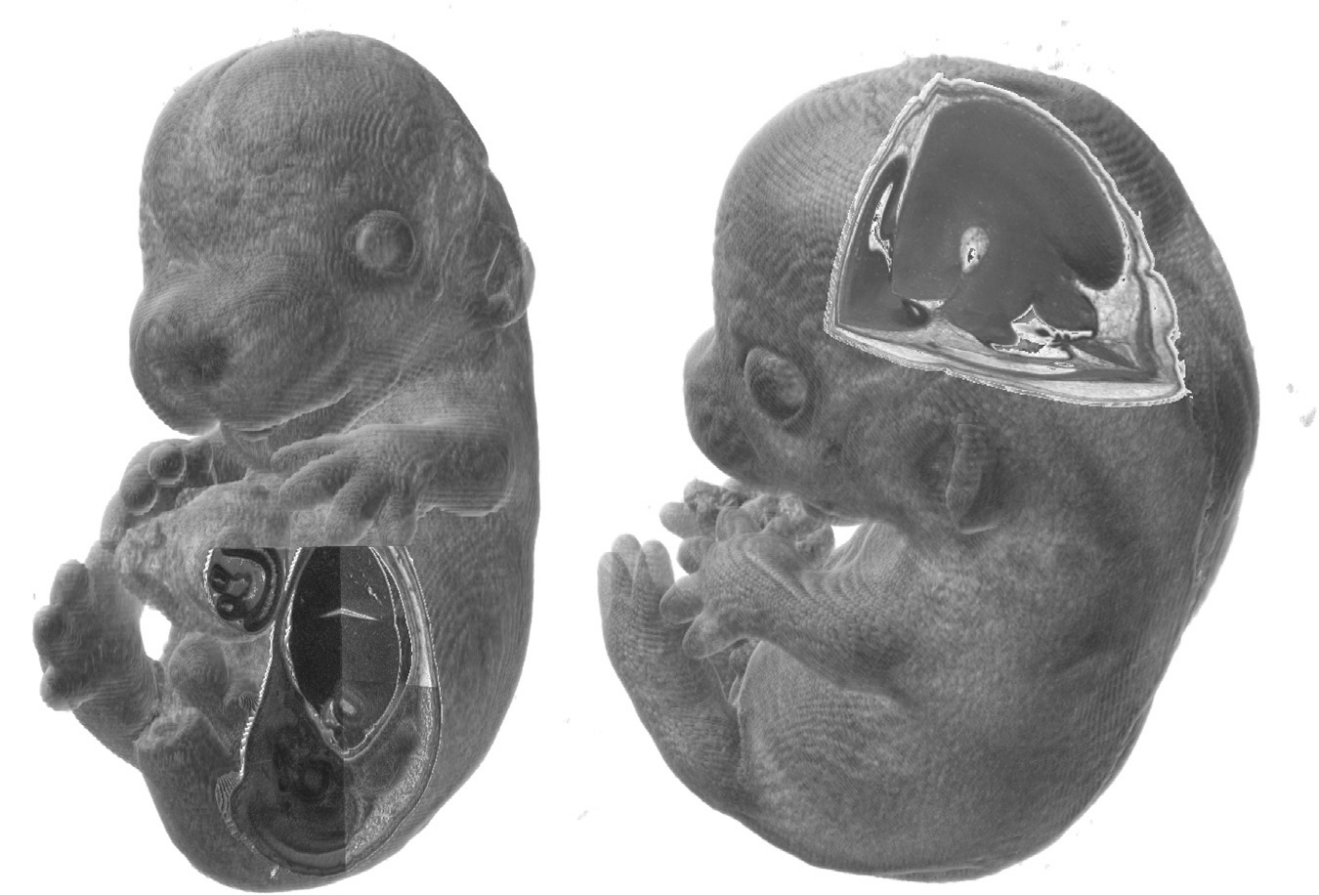
Two examples of 3D HREM data sets of mouse embryos at E14.5. Cut away sections show internal anatomy. Voxel resolution  3µ×3µ×3µ.

High-Resolution Episcopic Microscopy (HREM) gives three-dimensional isotropic images with excellent resolutions of 1–3 µm in all dimensions, as shown in Fig. 1 (Mohun and Weninger, 2012). The full set of HREM data used in this paper is available at http://data.mouseimaging.ca/HREM/. Such high resolution and homogenous isotropic data should allow, in principle, the identification of equivalent sections through multiple individuals. However, to date, such data sets have only been analyzed visually by going back and forth between individuals and looking at multiple slices to try to find equivalent microscopic features (Weninger et al., 2014).

This communication shows how to use relatively standard three-dimensional image processing tools to find equivalent high resolution microscopic sections enabling direct visual comparison and statistical analysis of similarities and differences within and between groups of individuals. Furthermore, within homogenous groups, the recognition of consistent structures over multiple individuals allows for the possibility of annotating finer levels of reproducible anatomy.

While this paper illustrates the method using mouse embryos, this ability to obtain homologous microscopic sections from multiple individuals should be of value for a variety of comparison tasks, at multiple stages of development, and in a variety of species.

**Fig. 2:**
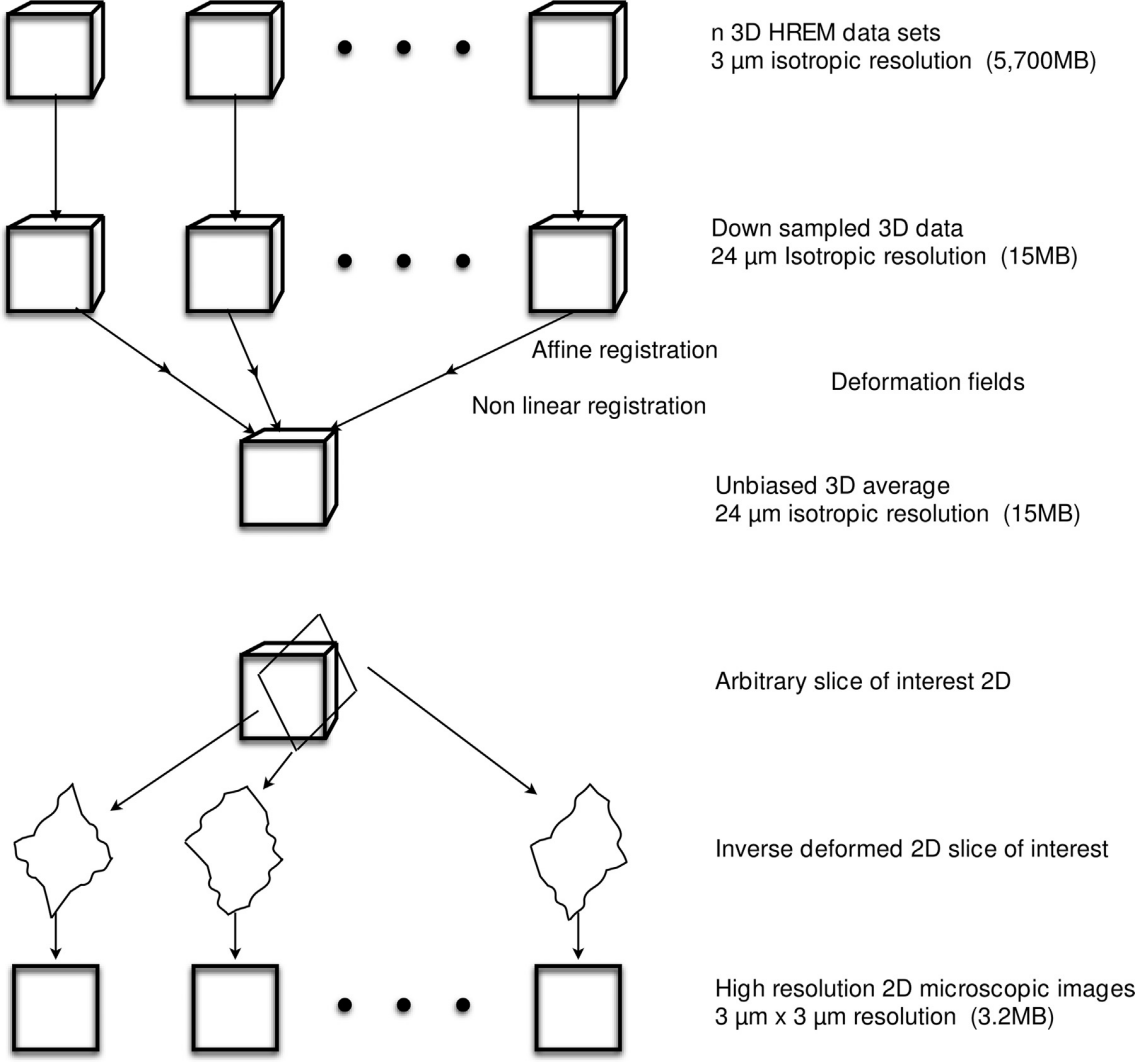
Schematic flow chart of the algorithm used in this paper.

**Fig. 3:**
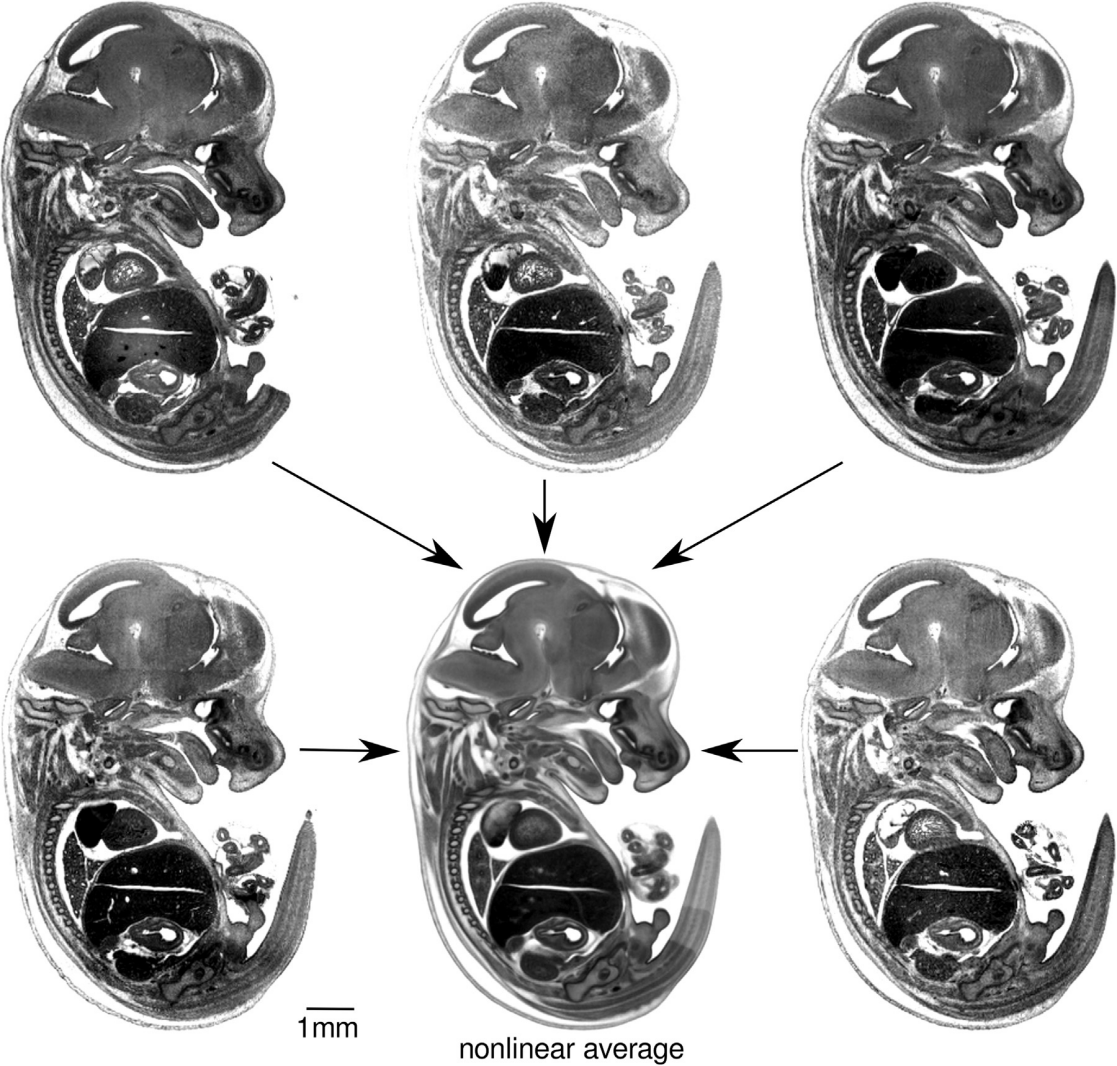
Slices from low resolution (down sampled from 3 µm isotopic resolution to 24 µm isotropic resolution) three-dimensional HREM data sets (N=5) nonlinearly registered together to yield an average image also at 24 µm resolution (centre), which shows clearer contrast and higher symmetry than any of the individual input images. The average image is comprised of 10 male and 11 female data sets at Theiler Stage 22 (and not just the five individuals illustrated in Fig. 3).

**Fig. 4:**
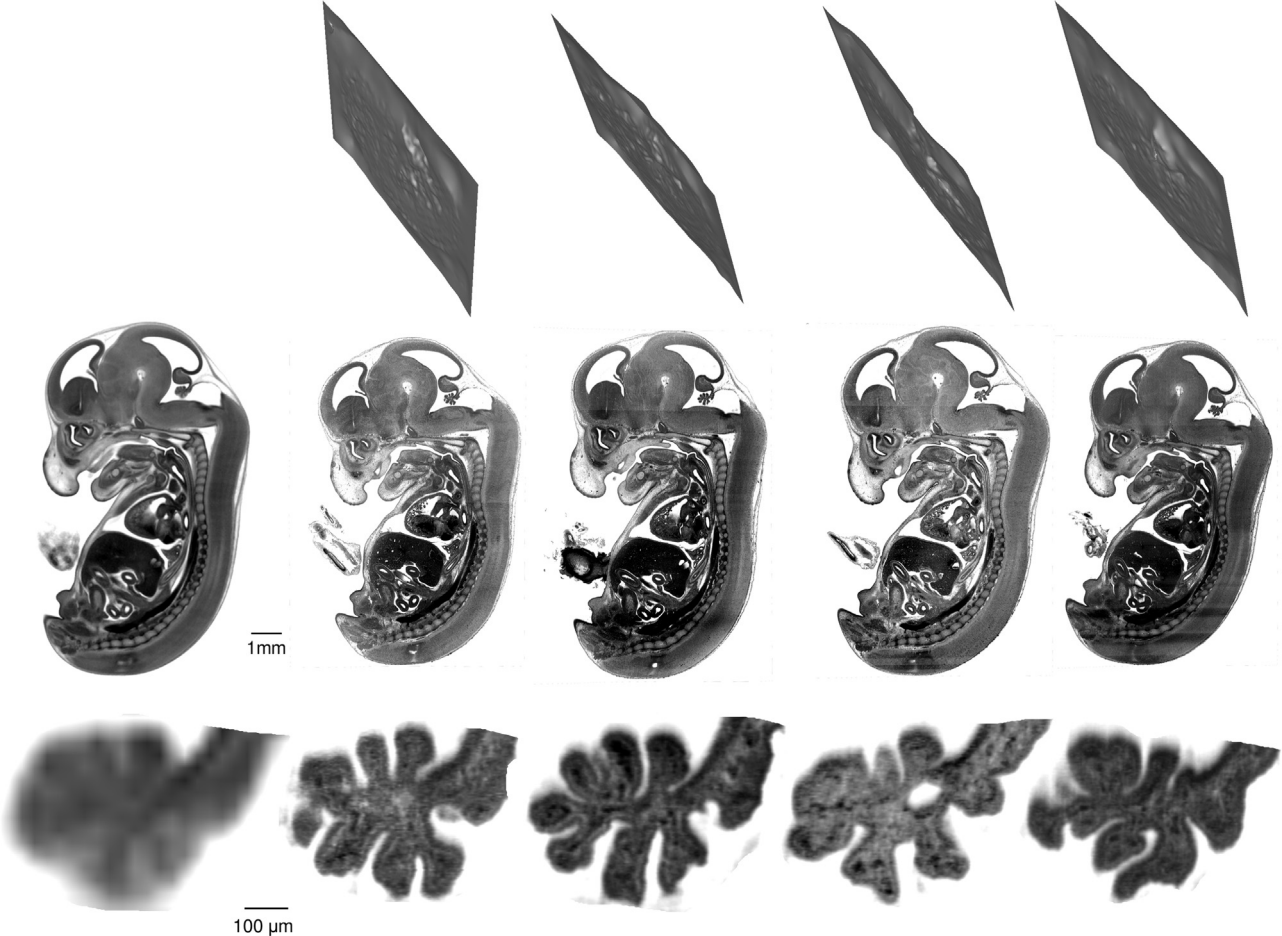
On the left is a sagittal slice through the low resolution (24 µm) average image which defines the target plane for the individual microscopic sections. On the right are data from four individual mouse embryos. The crumpled plans at the top show the effective “cut” plane through the three-dimensional high resolution (3 µm) HREM data that intersects all the points that are homologous to points in the average sagittal section. These “cut” planes are not planar because of the through plane components of the deformation fields required to bring the individual data sets into homologous registration in the average image. The middle row shows homologous microscopic high resolution (3 µm) images for four individual embryos. The lower row shows an enlarged segment of the region containing the choroid plexus.

A schematic in Fig. 2 summarizes the method. Down-sampling of the original high-resolution HREM data sets from 3 µm to 24 µm resolution presented no difficulty and the >500 reduction in the number of anatomical data points made the nonlinear registration of 21 embryos tractable (Fig. 3). Nonlinear registration followed algorithms that have been previously worked out for registration of microCT and MRI three-dimensional images of embryos and adult brains (Wong et al., 2012, Lerch et al., 2011). These algorithms are based on software developed at the Mouse Imaging Centre (MICe) (Friedel et al., 2014) and the Advanced Normalization Tools (ANTS) program (Avants et al., 2008) https://github.com/mouse-imaging-centre/pydpiper. Given the deformation fields required to bring each down-sampled individual HREM image into alignment with the average, the inverse fields are then used to back propagate any selected plane from the average three-dimensional image back into the individual HREM data, using linear interpolation to fill in intermediate points. Planes in any orientation can be selected for comparison. The interpolated deformation field is a best guess governed by the surrounding registration. The back propagated target plane, is then used to “cut” the original high resolution (3 µm) HREM data sets to yield homologous microscopic sections as shown in Fig. 4. These equivalent sections can be visually compared to evaluate consistency of the anatomy and to identify abnormal phenotypes in mutants without scrolling through complex three-dimensional data sets.

As resolution becomes finer, even in genetically identical groups of individuals, the concept of homologous anatomical regions eventually breaks down. This can be recognized in images of the vasculature which have similarities of general pattern in multiple individuals, but there is no possibility, beyond several branching levels, to identify points that are equivalent to each other in terms of spatial anatomy. The small vessel arborized vascular tree is essentially stochastic. Thus, detailed microscopic sections taken as close as possible through homologous regions allow one to identify anatomical patterns that are consistent over multiple individuals in a group and conversely, to also recognize parts of anatomy for which the patterning is essentially stochastic. Conventional published atlases are annotated on the basis of recognizing the same structure in microscopic sections of multiple individuals. However, where the anatomical patterning has become stochastic, specific names are no longer assigned.

**Fig. 5:**
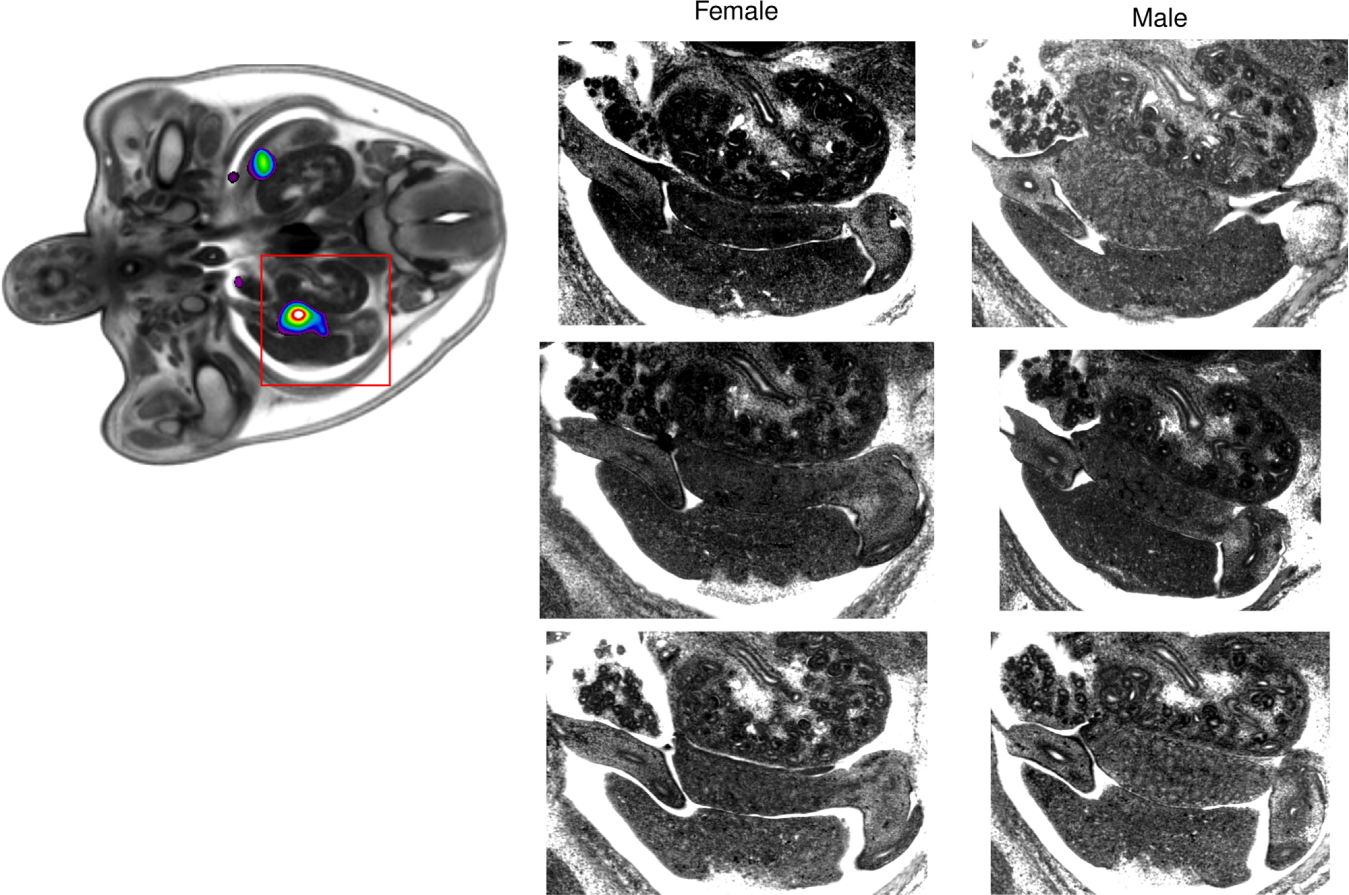
Quantitative assessment of volume differences identified from the Jacobian determinant of the individual deformation fields, automatically distinguish statistically significant differences between males and females in the region of the gonads. In turn, microscopic sections in homologous planes show the descending testes in the males which are absent in the females.

Beyond simple visual observation, the deformation fields and the associated Jacobians can be used to highlight statistically significant variation between groups of embryos as illustrated in Fig. 5 (Wong et al., 2014). Microscopic sections through the regions of inconsistent anatomy can then be automatically generated for visual confirmation.

The combination of three-dimensional isotropic data obtained with HREM with comparatively straight forward image processing techniques for registration of down-sampled images allow for the identification of homologous microscopic sections from multiple different individuals. This, in turn, enables direct visual comparison and various statistical analyses of anatomical sections at a microscopic resolution. Whether differences in anatomy between groups of individuals are identified by computer analysis techniques or just by visual observation, these methods allow for detailed microscopic comparison among many specimens at a cellular level with minimal spatial ambiguity.
